# Evaluation of clinically relevant serum proteins as biomarkers for monitoring COVID-19 severity, and end-organ damage among hospitalized unvaccinated patients

**DOI:** 10.1186/s12879-024-09113-6

**Published:** 2024-02-20

**Authors:** Mahetab R. Elhommosani, Masarra M. Sakr, Rania M. Abbas, Khaled M. Aboshanab

**Affiliations:** 1 Department of Microbiology and Immunology, Faculty of Pharmacy, Ain Shams University, Cairo, 11566 Egypt; 2 Department of Clinical Pathology, Faculty of Medicine, Ain Shams University, 11566 Cairo, Egypt

**Keywords:** COVID-19, Ferritin, Serum creatinine, Alanine transaminase, Aspartate transaminase, D-dimer, Procalcitonin, Interleukin-6, End-organ damage, Acute kidney impairment, Acute liver injury

## Abstract

**Background:**

The extensive variability and conflicting information in Coronavirus Disease 2019 (COVID-19) patient data have made it difficult for the medical community to gain a comprehensive understanding and develop clear, reliable guidelines for managing COVID-19 cases. As the world uncovers the diverse side effects of the pandemic, the pursuit of knowledge about COVID-19 has become crucial. The present study aimed to evaluate some clinically relevant serum proteins, providing analysis of the obtained results to employ them in the diagnosis, prognosis, and disease monitoring among COVID-19 patients.

**Methods:**

Samples were collected from 262 COVID-19 unvaccinated hospitalized patients. Measurement of certain serum proteins, namely C-reactive protein (CRP), ferritin, D-dimer, procalcitonin, interleukin-6 (IL-6), serum creatinine (SCr), alanine transaminase (ALT), aspartate transaminase (AST) was done using standard methods. Statistical analysis was performed on the obtained data and the results were correlated to the severity and prognosis.

**Results:**

The calculated Mortality rate was found to be 30% with a higher percentage observed among females. The results showed elevation in serum CRP, ferritin, D-dimer, and procalcitonin in most of the patients, also some patients had elevated SCr, ALT, and AST levels indicating end-organ damage. The statistical analysis displayed a strong correlation between serum levels of CRP and ferritin, between D-dimer and ferritin, and between ferritin and procalcitonin. No significant difference was observed between male and female patients’ serum levels of the tested serum proteins. A significant correlation between increased serum procalcitonin and mortality was observed.

**Conclusion:**

The levels of measured serum proteins were impacted by SARS-CoV-2 infection. Serum ferritin, CRP, D-dimer, and procalcitonin are good predicting tools for end-organ damage and acute kidney impairment in COVID-19. Procalcitonin is a strong indicator of severity and mortality in hospitalized COVID-19 patients.

## Background

The Coronavirus disease 2019 (COVID-19) has been a massive challenge for the world, and we still have many questions about this disease and how to treat it. COVID-19 is an infectious respiratory disease caused by Severe Acute Respiratory Syndrome Coronavirus-2 (SARS-CoV-2), a single-stranded (positive sense) RNA virus [1]. In the Summer of 2023, more than 1.5 million cases of COVID-19 were confirmed, and over 2500 deaths within 28 days according to the WHO weekly epidemiological update of August 2023 [2]. The disease was first identified in late 2019 in Wuhan, China, and since then it has spread pandemically all over the world, causing a great ordeal to the local and international health authorities trying to contain it [3].

Symptoms vary from one person to another but mainly include cold-like symptoms (malaise, fever, headache), cough, sore throat, and chest CT changes, some may develop gastrointestinal symptoms such as vomiting and diarrhea [4]. Other patients may develop acute respiratory distress syndrome (ARDS) which is a serious condition with a significant rate of mortality that needs hospitalization and possibly supplemental oxygen [5, 6]. Confirmatory diagnosis is done by RT-PCR to identify the viral genome in a nasopharyngeal swab, although false negative results are probable [5].

The high variation and contradiction in COVID-19 patients’ information have rendered the medical community unable to fully comprehend and manage COVID-19 cases with clear, trustful guidelines. As the world explores the varied consequences of the pandemic, the quest for knowledge concerning COVID-19 has become essential. The interactions between SARS-CoV-2 and the diverse biochemical markers offer valuable insights into the disease’s pathogenesis and possible treatment pathways [7]. Serological tests have been used to aid in the diagnosis, prognosis, and course of treatment [8] including C-reactive protein (CRP), D-dimer, Ferritin, interleukin 6, Alanine transaminase (ALT), Aspartate transaminase (AST), and serum creatinine. These biomarkers reflect various aspects of inflammation, organ damage, coagulation, and immune response, consequently, they have been used as critical indicators in the clinical management of COVID-19 patients [9].

The re-spreading and emergence of new variants require a vast number of reliable studies to further enhance the scientific community’s perception and predictability of the disease and its various outcomes. Therefore, the objective of this study was to examine the levels of these serological biomarkers in hospitalized unvaccinated COVID-19 patients and to provide a better understanding of the relationship between the different test results and COVID-19 prognosis and some of the clinical-relevant findings in COVID-19 patients, such as kidney and liver functions represented by SCr, ALT, and AST, respectively.

## Materials and methods

### Study design and participants

This study was performed on patients admitted to Ain Shams University hospitals from January 2022 to June 2022 older than 18 years of age (> 18) with confirmed COVID-19 diagnosis via RT-PCR on nasopharyngeal or oropharyngeal swabs, excluding patients with negative RT-PCR, younger than 18 years, with known history of kidney or liver diseases or vaccinated patients. This research underwent a review process and received approval from the Ethics Committee Faculty of Pharmacy at Ain Shams University, Cairo, Egypt (ACUC-FP-ASU RHDIRB2020110301 REC # 34]. The experiment was considered to pose no additional risk to patients, allowing it to be conducted under a waiver of informed consent. This study adhered to the Declaration of Helsinki and followed all relevant local and national regulations.

### Sample collection and analysis

#### Sample collection

All samples were collected from COVID-19 patients upon admission to Ain Shams University isolation hospital. A 10 mL venous blood sample was collected from each patient and centrifuged at 2000 rpm for 15 min, by Rotofix 32 A (Hettich, Germany).

### Measurement of serum proteins

#### Measurement of ferritin, interleukin-6 and procalcitonin

Three mL of blood were added to a gel tube to determine ferritin, IL-6, and procalcitonin by electrochemiluminescence immunoassay on Cobas e 411 (Roche, Basel, Switzerland).

#### Measurement of D-dimer

Another 2 mL was added to a citrate tube containing sodium citrate as an anticoagulant to obtain plasma to determine D-dimer by automated chromogenic assay on Sysmex XN-1000 (Sysmex Corporation, Kobe, Japan).

#### Measurement of ALT, AST, and SCr

Five mL of the collected sample were added to a gel tube containing inert acrylic gel and clot activator to separate cells from serum- to determine ALT, AST, and SCr By automated spectrophotometric assay on AU680 (Beckman Coulter, Tokyo, Japan).

### Statistical analysis

Descriptive statistics such as the mean value ± the standard error of the mean (Mean ± SE ), the standard deviation (StDev), Minimum, the first quartile where 25% of values are below (Q1), Median, the third quartile where 75% of values are below (Q3), Maximum, and IQR Interquartile Range, It is the range between the first quartile (Q1) and the third quartile (Q3) and represents the middle 50% of the data, were calculated for continuous variables. While categorical variables were represented as contingency tables displaying the counts and percentages. Univariate analysis to assess relations between different variables was performed using Pearson’s Chi-square test. A Student’s t-test was used to compare parameters including only gender.

## Results

### Patient demographics

A total of 262 patients were included in this study, of which there were 131(50%) women and 131 (50%) men, their mean age was 48.034 ± 0.855 with the youngest being 18 and the oldest 79. There were 78 deaths with a mortality rate of 30%, 31 of them were males (40%) and 47 were females (60%).

### Overall descriptive statistics for the biomarkers

Results displayed in Table 1 show an elevation in most patients’ serum levels of CRP, Ferritin, D-dimer, and procalcitonin. Results displayed higher levels in D-dimer values in patients who eventually died where the average recorded serum D-dimer level in patients who survived was 2.85 vs. an average serum level of 6.68 in fatalities. Also, a great segment of the COVID-19 patients had elevated SCr levels and to a lesser extent ALT and AST levels.

**Table 1:** Descriptive statistics of all biomarkers

Variable	Mean ± SE	* StDev *	Minimum	Q1	Median	Q3	Maximum	IQR
Age	48.034 ± 0.855	13.834	18	40	50	58	79	18
CRP	140.3 ± 29.5 mg/L	471.6	0	33.6	78.9 mg/L	162	7402 mg/L	128.4
SCr	1.5188 ± 0.0881 mg/dL	1.4204	0.2 mg/dL	0.8	1 mg/dL	1.6	8.9 mg/dL	0.8
ALT	62 ± 10.3 IU/L	163.2	13 IU/L	20	30 IU/L	54	1799 IU/L	34
AST	72.2 ± 17.4 IU/L	276	7 IU/L	22	34 IU/L	54	3775 IU/L	32
Ferritin	1144.4 ± 94 ng/mL	159.7	34 ng/mL	343.7	740.4 ng/mL	1337.6	10,000 ng/mL	994
Procalcitonin	1.29 ± 0.309 ng/mL	4.827	0.01 ng/mL	0.06	0.176 ng/mL	0.875	51.24 ng/mL	0.815
D-dimer	3.314 ± 0.268 µg/ mIFEU	4.278	0	0.72	1.67 µg/ mIFEU	3.82	20 µg/ mIFEU	3.1
IL-6	100.3 ± 38.9 pg/mL	487.9	0.9 pg/mL	6.6	16.8 pg/mL	38.5	5000 pg/mL	31.9

Descriptive statistics including: mean, standard error of the mean, minimum value, median, maximum value, and interquartile range for the measured biomarkers

### Gender-associated differences in biomarkers levels

**Fig. 1:**
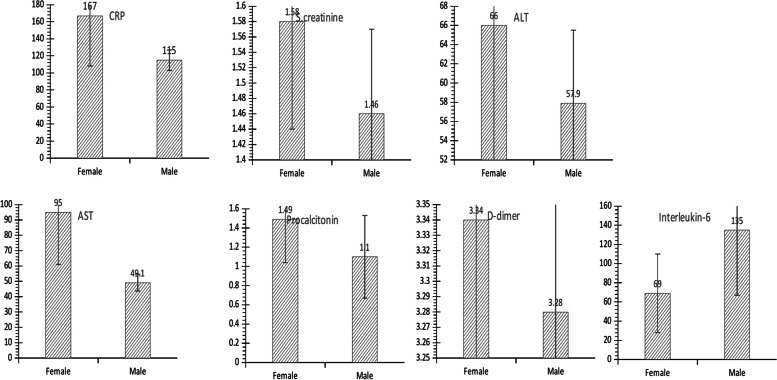
Gender-associated differences in biomarkers levels. Column graphs displaying the relationship between the male against female serum levels of CRP, SCr, ALT, AST, procalcitonin, D-dimer, and interleukin-6 in COVID-19 hospitalized unvaccinated patients. Showing some differences but no statistically significant differences in a student’s T-test analysis

Using independent Student’s T-test statistics to determine if there was a significant difference between male and female patients’ serum protein levels, while the null hypothesis is that there was no statistical difference between males and females and the alternate hypothesis is that there was a significant difference between the two groups, at a significant *P*-value < 0.05, it was found that there was no significant difference between male and female patients’ level of CRP, SCr, ALT, AST, Ferritin, D-dimer, Procalcitonin or IL-6 as shown in Table 2 and represented in Fig. 1.

**Table 2:** T-test of gender vs. all biomarkers

Variables	Male, mean ± SE	Female mean ± SE	Male StDev	Female StDev	*P*-Value
CRP	115 ± 12 mg/L	167 ± 59 mg/L	132	660	0.385
SCr	1.46 ± 0.11 mg/dL	1.58 ± 0.14 mg/dL	1.27	1.56	0.477
ALT	57.9 ± 7.6 IU/L	66 ± 19 IU/L	85.2	215	0.692
AST	49.1 ± 5.5 IU/L	95 ± 34 IU/L	61.7	384	0.186
Ferritin	1308 ± 149ng/mL	978 ± 112 ng/mL	1702	1271	0.078
D-dimer	3.28 ± 0.35 µg/ mIFEU	3.34 ± 0.41 µg/ mIFEU	3.98	4.58	0.912
Procalcitonin	1.1 ± 0.43 ng/mL	1.49 ± 0.45ng/mL	4.78	4.89	0.532
Interleukin-6	135 ± 68 pg/mL	69 ± 41 pg/mL	587	376	0.409

The results of independent student T-test to compare the means of the male group and female group in the measure biomarkers

### Correlation between COVID-19-associated serum proteins

**Fig. 2:**
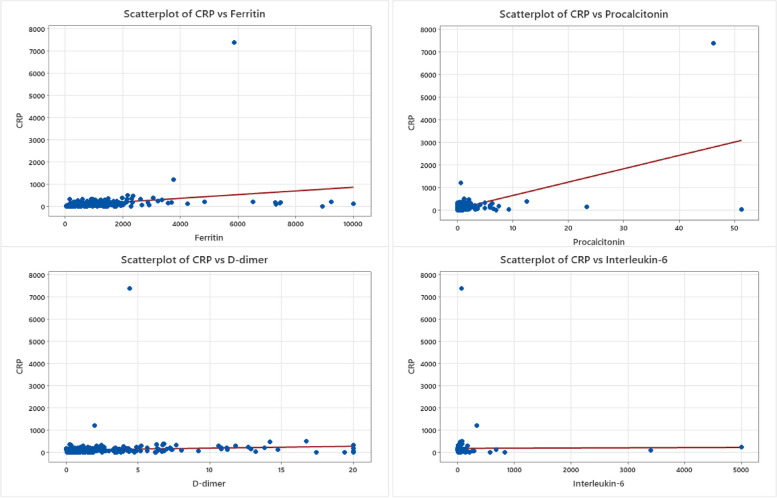
CRP relationship to other inflammatory biomarkers. Scatterplot graphs of CRP levels against ferritin levels, CRP levels against procalcitonin levels, CRP levels against D-dimer levels, and CRP levels against interleukin-6 levels in COVID-19 hospitalized unvaccinated patients, showing a significant relationship between CRP against ferritin

**Fig. 3:**
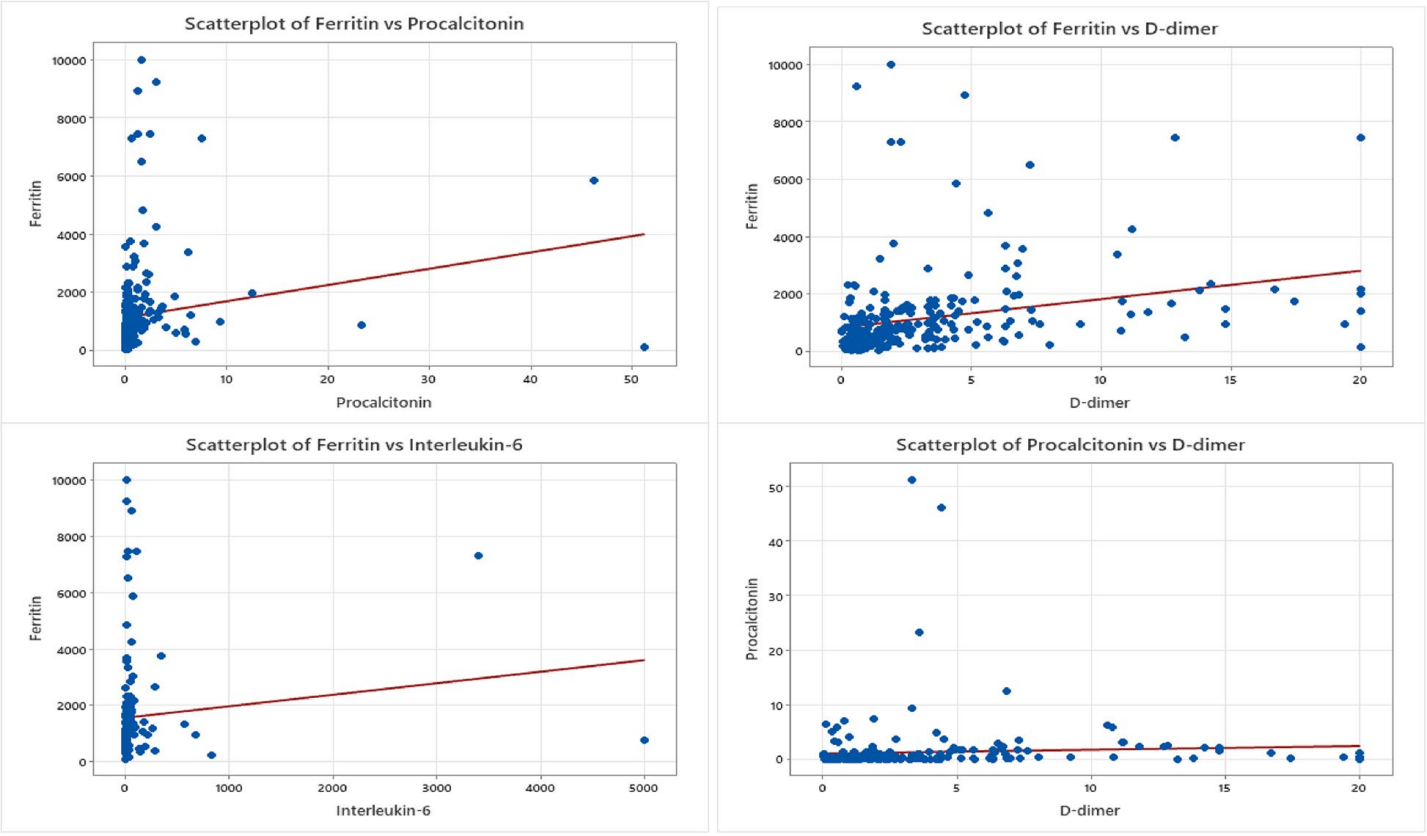
Ferritin and D-dimer relationship to Procalcitonin and IL-6. Scatterplot graphs displaying the rest of inflammatory biomarkers relationships including ferritin levels against procalcitonin, ferritin levels against D-dimer levels, ferritin levels against interleukin-6 levels, and procalcitonin levels against D-dimer levels in COVID-19 hospitalized unvaccinated patients, with only two significant relationships which are ferritin levels against procalcitonin and ferritin levels against D-dimer

**Fig. 4:**
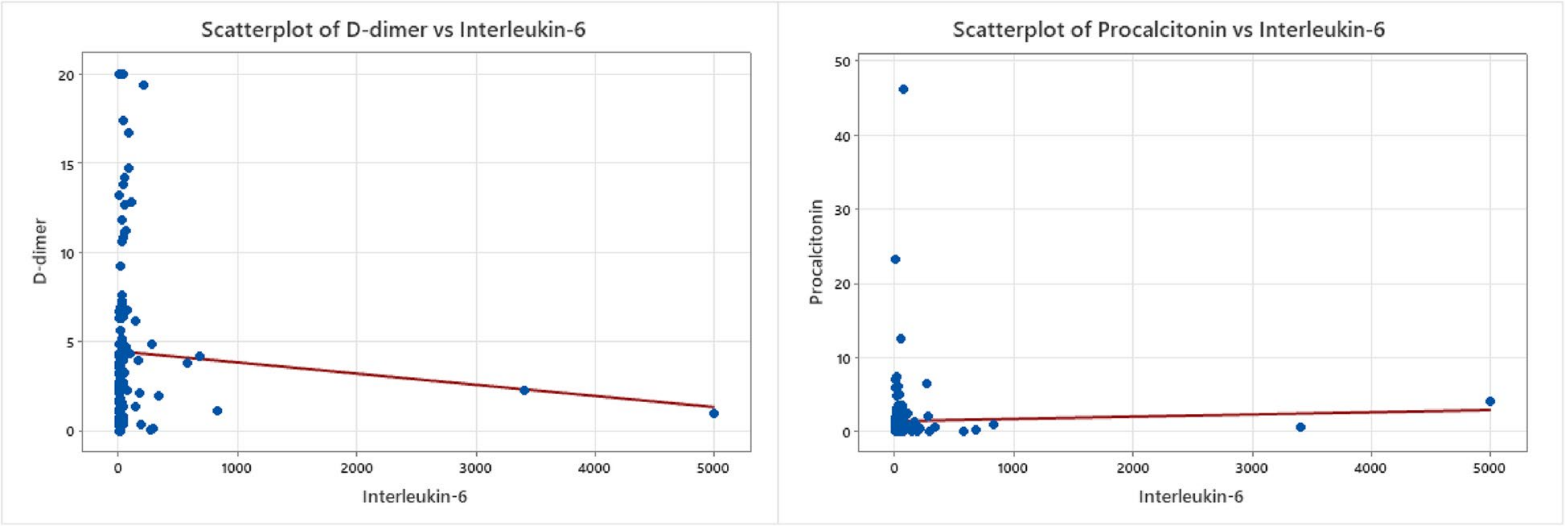
IL-6 relationship to D-dimer and Procalcitonin. Scatterplot graphs of two inflammatory biomarkers relationships, D-dimer levels against interleukin-6 in COVID-19 hospitalized unvaccinated patients and the statistically significant relationship of procalcitonin against interleukin-6

Various inflammatory indicators and biomarkers are detailed in Table 3. The Chi-square test findings, as illustrated in Fig. 2, demonstrate a robust direct relationship between C-reactive protein (CRP) and Ferritin. Additionally, noteworthy significant relationships were identified, including the correlations between Ferritin and both procalcitonin and D-dimer, each exhibiting a *P*-value below 0.05 as shown in Fig. 3. The other noteworthy discovery was the highly significant association between Procalcitonin and IL-6, with a *p-value* of 0.001 which is displayed in Fig. 4.

**Table 3:** Correlation between various inflammatory indicators and biomarkers

Correlation: a. *P*-value as categorical high and normal level	CRP			
Ferritin	0.00021	Ferritin		
D-dimer	0.278	0.039	D-dimer	
Procalcitonin	0.054	0.00014	0.053	Procalcitonin
Interleukin-6	0.287	0.481	0.725	0.001

The *p*-value of Pearson’s chi square test used to explore the correlation between the biomarkers

### Relationship between liver enzymes and kidney function test and other tested biomarkers

**Fig. 5:**
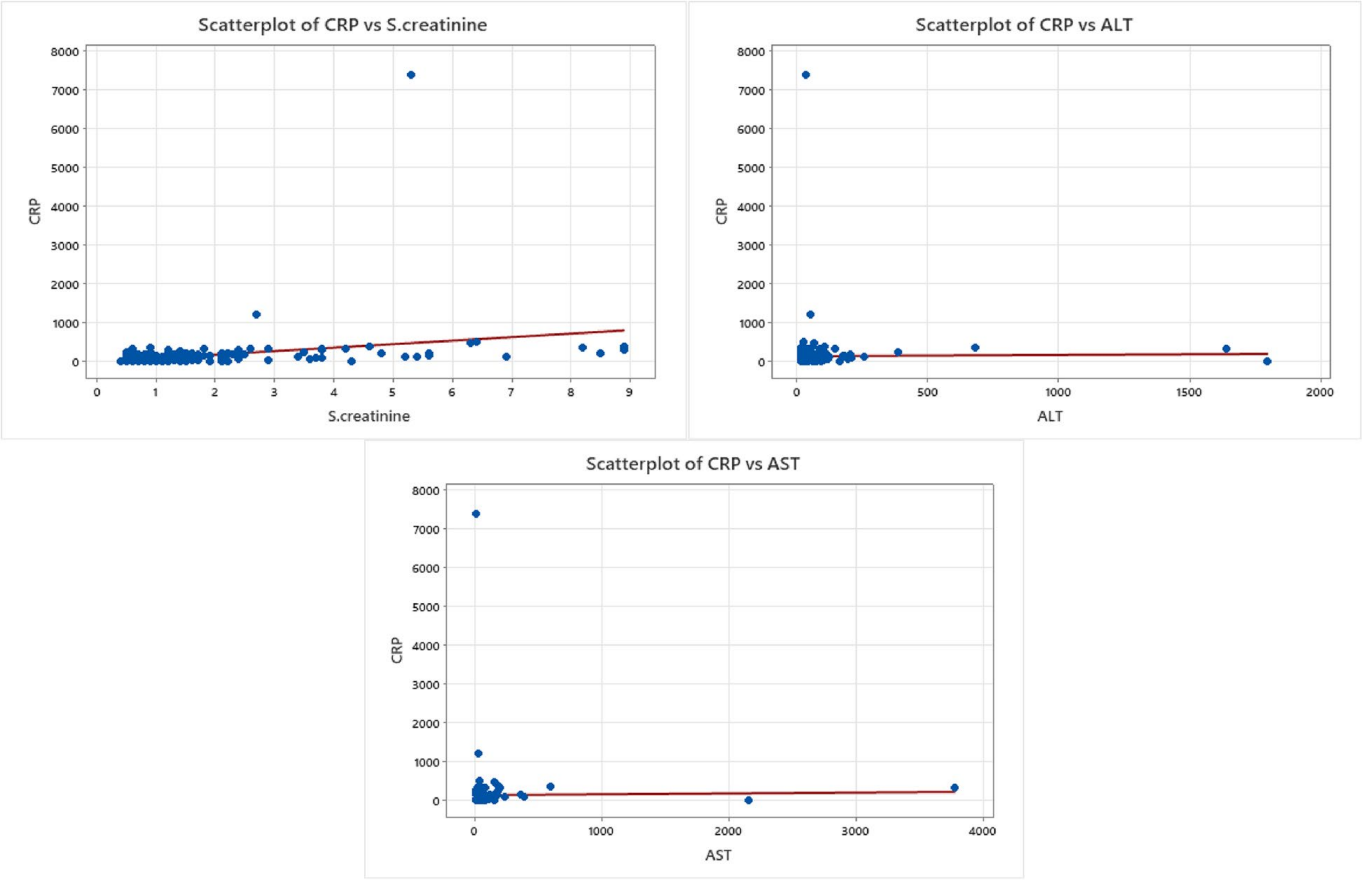
CRP relationship to SCr and liver enzymes. Scatterplot graphs of CRP levels against SCr levels, CRP levels against ALT, and CRP levels against AST in COVID-19 hospitalized unvaccinated patients, showing a significant relationship between CRP levels against SCr levels

**Fig. 6:**
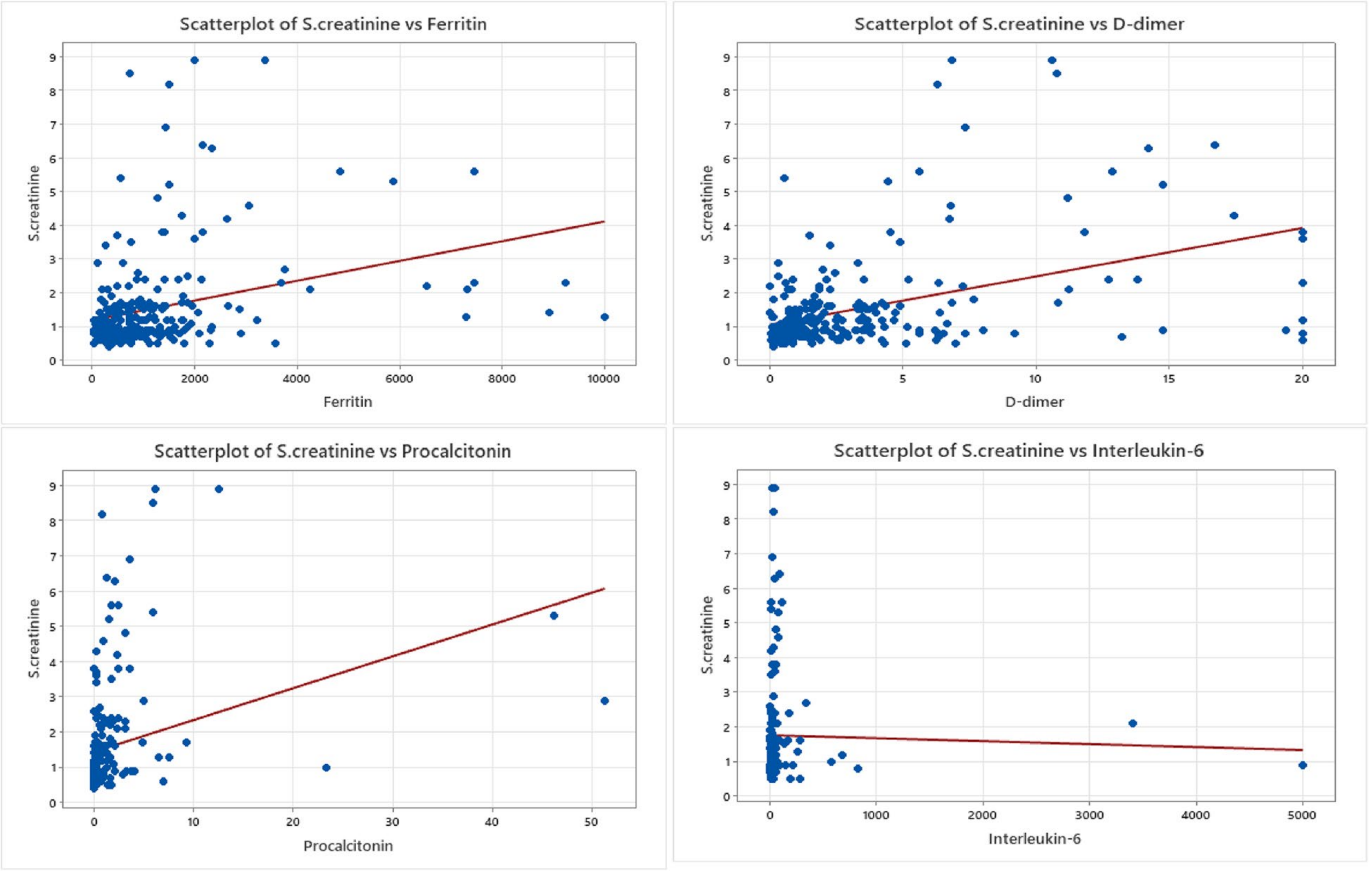
SCr relationship to COVID-19 inflammatory biomarkers. Scatterplot graphs of SCr levels against the rest of inflammatory biomarkers in COVID-19 unvaccinated patients including a significant relationship between SCr levels against ferritin levels, another significant relationship between SCr levels against D-dimer levels, and a significant relationship between SCr levels against procalcitonin levels, while the relationship between SCr levels against interleukin-6 levels is statistically not significant

**Fig. 7:**
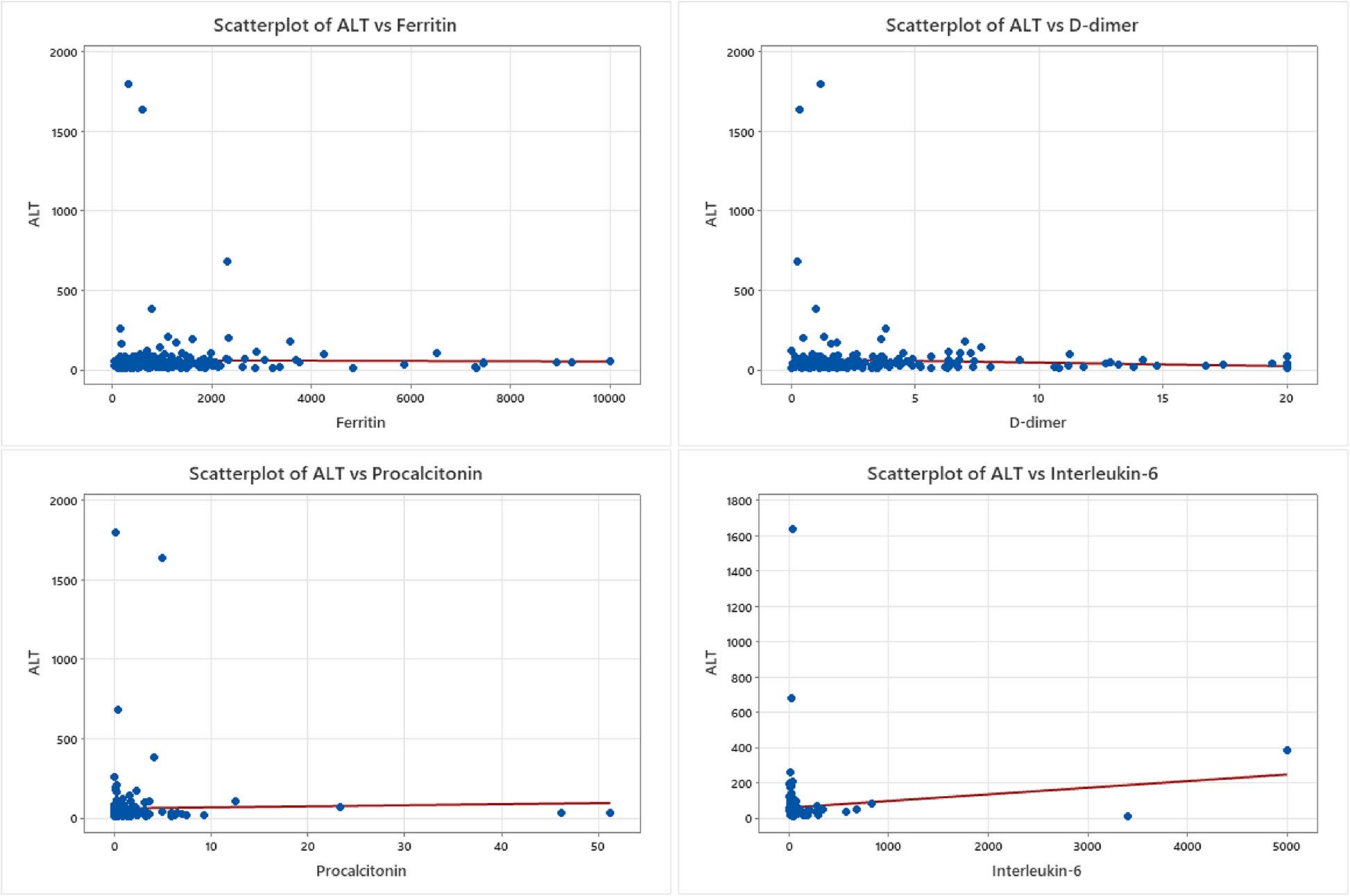
ALT relationship to COVID-19 biomarkers. Scatterplot graphs showing ALT levels’ relationship to Ferritin, D-dimer, procalcitonin, and interleukin-6 levels in COVID-19 hospitalized unvaccinated patients with no statistical significance

**Fig. 8:**
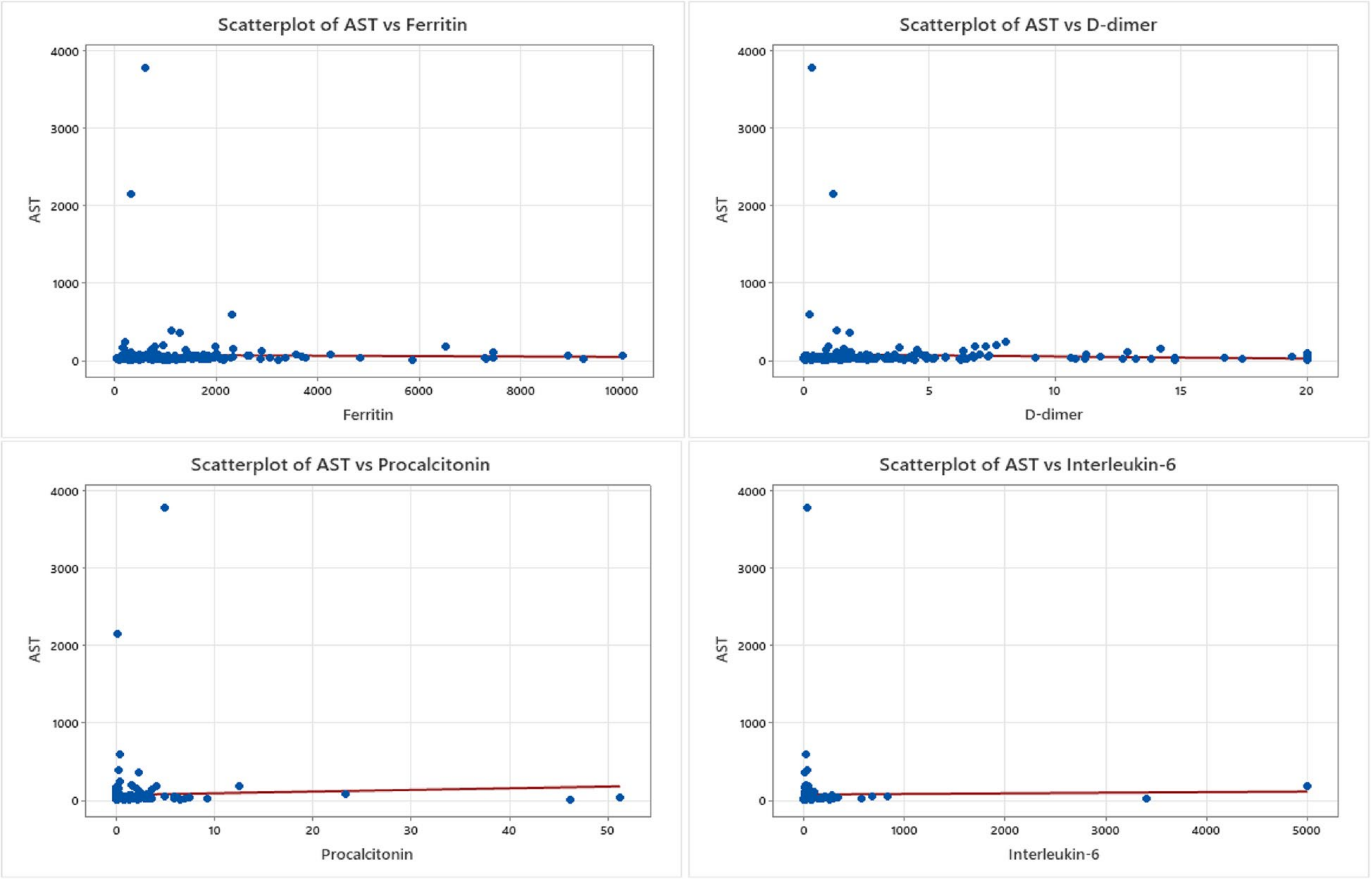
AST relationship to COVID-19 biomarkers. Scatterplot graphs of AST levels against ferritin, D-dimer, procalcitonin, and interleukin-6 levels in COVID-19 hospitalized unvaccinated patients, with no statistically relevant relationships

The results presented in Table 4 highlighted an evident correlation between CRP and serum SCr as demonstrated in Fig. 5. Furthermore, our findings revealed a substantially significant relationship between SCr and serum. ferritin, D-dimer, and procalcitonin as shown in Fig. 6. There were no statistically significant relationships observed between alanine transaminase (ALT) or aspartate transaminase (AST) and any of the COVID-19 biomarkers under investigation that we displayed in Figs. 7 and 8.

**Table 4:** Relationship between tested biomarkers and liver enzymes and Serum creatinine

Correlation: *P*-value	SCr	ALT	AST
CRP	0.028	0.139	0.675
Ferritin	0.00023	0.194	0.161
D-dimer	0.001	0.259	0.184
Procalcitonin	0.000175	0.584	0.148
IL-6	0.101	0.834	0.064

The *p*-value of Pearson’s chi square test used to explore the correlation between the biomarkers

### Relationship between procalcitonin level, d-dimer level and mortality rate

**Fig. 9:**
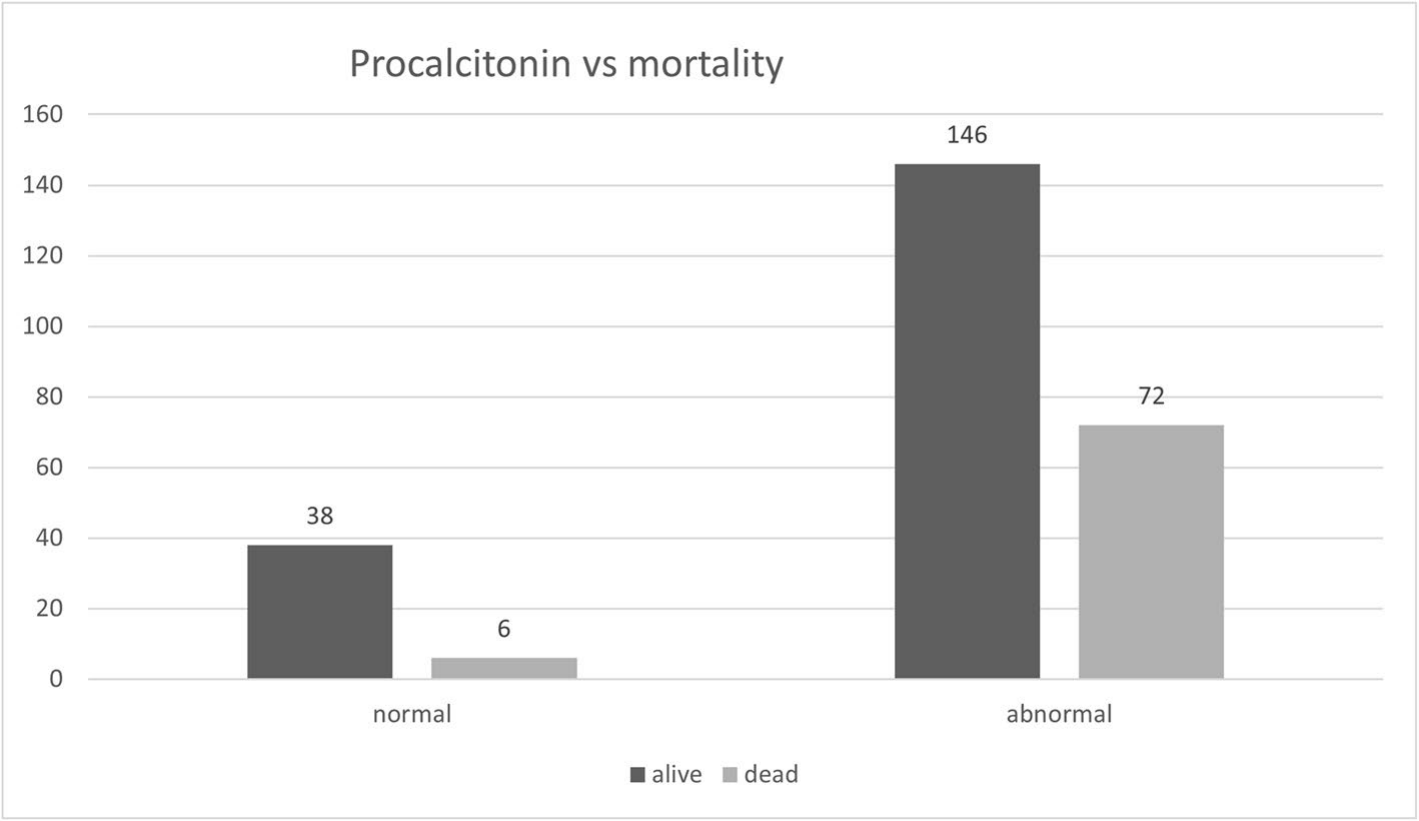
Mortality relationship to Procalcitonin. A column chart showing the relationship of procalcitonin levels represented as normal and abnormal levels (elevated procalcitonin level) to mortality in hospitalized unvaccinated COVID-19 patients

Using contingency analysis, procalcitonin was found to have a positive relationship to mortality rate with a *p*-value = 0.0102 (Table 5; Fig. 9). The average D-dimer level of the survived patients was found to be 2.017, while the average D-dimer level of the non-survived patients was found to be 5.184. However, the relationship of D-dimer level elevation and mortality rate was found non-significant with *p*-value = 0.582 (Table 6).

**Table 5:** Contingency table of mortality against procalcitonin levels in COVID-19 patients

	Alive	Dead	Sum
Normal	38 (14.5%)	6 (2.3%)	44
Abnormal	146 (55.5%)	72 (27.5%)	218
			Grand total: 262

**Table 6:** Contingency table of mortality against D-dimer levels in COVID-19 patients

	Alive	Dead	Sum
Normal	36 (13.74%)	13 (4.96%)	49
Abnormal	148 (56.49%)	65 (24.81%)	213
			Grand total: 262

## Discussion

As the prevalence of various COVID-19 variants continues to rise, a persistent examination of COVID-19-related serum proteins as both diagnostic and prognostic tools has become increasingly crucial as it serves as an essential strategy in staying ahead of the ever-changing nature of COVID-19, providing valuable insights that contribute to more effective diagnostic and prognostic tools in the face of emerging variants. In our study, we managed to screen a total of 262 hospitalized unvaccinated patients with confirmed COVID-19 infection for several serum proteins (CRP, Ferritin, D-dimer, Procalcitonin, IL-6, SCr, ALT, and AST). A mortality percentage of 30% was recorded which is possibly high since they were hospitalized and unvaccinated. Results in our study come in accordance with a previous study in Jordan which reported a death rate of 37% among hospitalized COVID-19 patients [10]. It is worth noting here that some previous studies mentioned that ethnic variability plays an important role in the mortality rate of COVID-19 infection [11], yet little has been reported about Middle Eastern ethnicity so far. Gender comparison of fatality rate revealed that 40% of the dead patients were males while 60% were females which is contradictory to some studies which state that the female surviving rates are higher than the males as in a study by Nguyen et al. [12]. On the other hand, another study explained that gender-associated mortality statistical analysis could be misleading as it mainly reflects the first few weeks of the pandemic infection and hospitalization surge [13].

Results in our study showed that there was a trend increase in the tested biomarkers in all the patients. This comes in accordance with several studies that established the increase in inflammatory biomarkers in COVID-19 infection [7, 14]. CRP, a liver-produced protein that mainly indicates an infection, inflammation, or tissue injury, was elevated in almost all patients with a mean value of 140 mg/L while its normal blood level is < 10 mg/L. Similarly, ferritin, a serum protein that increases inflammation and is strongly associated with COVID-19 infection, was elevated in most patients. Procalcitonin, a precursor protein to the calcitonin hormone that is produced by various cells in the body, primarily by certain cells in the thyroid gland, normally has a very low serum level. However, procalcitonin levels can rise significantly in response to bacterial infections, and viral infections as in COVID-19 patients’ cases [15]. The measured procalcitonin levels in this study were elevated in certain patients indicating severe viral infection or bacterial infection or even possibly sepsis [16].

Another studied serum protein was the D-dimer, a degradation product of fibrin indicating hypercoagulopathy and considered a key biomarker for ruling out venous thromboembolism [17]. Hyper-coagulopathy is a very concerning complication of COVID-19, which causes both microthrombi in smaller blood vessels and large blood vessel thrombi like deep vein thrombosis and pulmonary embolism [18, 19]. This increase in thrombotic events is mainly due to the massive hyperinflammatory response, inflammatory endothelial injury, and hypoxemia which increases reactive oxygen species (ROS) formation [20]. Results in our study showed that levels of D-Dimer were raised in many patients. Although the contingency test showed no significant correlation between increased D-dimer levels and mortality, the increase in D-dimer level was notably higher in patients who died where the average D-dimer value was 2.3 times higher than the average D-dimer value in alive patients. We also found a robust correlation between D-dimer as an indicator of hypercoagulopathy in COVID-19 patients and serum ferritin which signals an inflammatory response. We noticed that patients with higher ferritin levels had higher D-dimer levels, which put them at increased risk of developing thrombotic events. This comes in agreement with several previous studies that reported that higher D-dimer levels were observed in patients with severe COVID-19 disease and which showed the importance of D-dimer level monitoring in the guidance of COVID-19 anticoagulant treatment strategy [18].

We also measured the levels of Interleukin-6, a pro-inflammatory cytokine, responsible for the release of CRP during the acute phase response to inflammation [21]. Il-6 was reportedly increased during what is called a cytokine storm in COVID-19 disease [22]. In this study, we found a significant correlation between the increase in serum IL-6 levels and the increase in procalcitonin levels which indicates severe inflammation due to COVID-19 infection and confirms the importance of monitoring IL-6 levels during COVID-19 treatment. Previous studies have reported elevated IL-6 levels as a sign of severe COVID-19 infection [23] and lately, studies have focused on the possibility of IL-6 inhibitors to serve as a treatment in cytokine storm syndrome as tocilizumab (actemra®), sarilumab and siltuximab [24].

As for gender-related differences in biomarkers levels, no statistically significant differences were found in the levels of CRP, SCr, ALT, AST, Ferritin, D-dimer, Procalcitonin, or IL-6 between male and female patients in this study on contrary to a study by Schully et al. [25] that stated the increased levels of ferritin, CRP, and IL-6in males more than in females.

Regarding end organ damage, biomarkers like SCr as well as ALT and AST, the liver function enzymes were assessed. Increased levels of SCr as well as ALT and AST were observed in some patients in this study. This finding indicates COVID-19’s association to end organ damage and targeting other organs besides the lungs as kidney and liver, as established by multiple studies evaluating Acute kidney injury (AKI) in COVID-19 infections and acute liver injury [26, 27].

Kidney involvement and acute kidney injury commonly reported in COVID-19 hospitalized patients could be attributed to the immense inflammation and cytokine storm caused by SARS-COV-2, the thrombotic microangiopathy, and formation of clots in the nephron, hypoxia, hypoperfusion, and also the direct effect of the virus on renal cells and its renal tropism [28]. In this study, we have found a significant statistical correlation between high levels of SCr denoting AKI and serum ferritin, CRP, procalcitonin, and D-dimer levels in those patients which confirms the previous explanations of the association of inflammation and hypercoagulopathy in the events of kidney injury in COVID-19 Patients. A previous study on 41 patients with chronic kidney disease found that patients with higher levels of CRP, ferritin, and D-dimer were at higher risk of developing AKI than patients with lower levels of these biomarkers [26]. The results of our study support this statistical finding.

Although acute liver injury has also been reported in COVID-19 cases due to inflammation, cytokine storm, hypoxia, hypoperfusion, and direct cytopathic effect [29], there has been no significant statistical relation between ALT and AST levels as liver function enzymes and any other inflammatory biomarker measured in our study which in turn supports the hypoxia and direct cytopathic effect due to viral tropism to angiotensin-converting enzyme 2 (ACE2) receptors in liver cells as the probable causes of acute liver impairment. Some previous studies reported that procalcitonin is a key biomarker that serves as a major indicator of the disease’s prognosis and severity [30, 31] also, another study indicated that procalcitonin is directly linked to lung tissue damage and hyperinflammatory response [32]. Our statistical analysis also revealed a strong correlation between increased serum procalcitonin levels in COVID-19 patients and increased mortality in this group. Likewise, an observational study of 271 patients in New York in 2021 found that procalcitonin could be linked directly to increased mortality and intensive care unit admission [15] which qualifies procalcitonin to be an important indicator for the severity of the disease and mortality.

## Conclusions

Serum proteins like C-reactive protein, ferritin, D-dimer, procalcitonin, interleukin-6, serum creatinine, ALT, and AST remain to be promising tools for monitoring the prognosis and predicting the complications of COVID-19. The statistical correlations of the serum proteins obtained in this study represent a potential tool in developing a novel prognostic COVID-19 scoring system that also incorporates and predicts acute kidney impairment as a side effect of COVID-19, additionally using procalcitonin levels as an indicative tool of mortality in COVID-19 hospitalized patients. IL-6 correlation to procalcitonin could be of great value in anticipating cytokine storm syndrome that needs further investigation.

## Data Availability

All data generated or analyzed during this study are included in this published article in the main manuscript.

## Abbreviations

COVID-19: Coronavirus disease 2019
CRP: C-reactive protein
SCr: Serum creatinine
IL-6: Interleukin-6
ALT: Alanine transaminase
AST: Aspartate transaminase
SARS-CoV-2: Severe acute respiratory syndrome coronavirus 2
AKI: Acute kidney impairment
ALI: Acute liver injury
RNA: Ribonucleic acid
CT: Computed tomography
ARDS: Acute respiratory distress syndrome
RT-PCR: Real-time polymerase chain reaction
StDev: Standard deviation
Q1: First quartile
Q3: Third quartile
IQR: Interquartile range
ROS: Reactive oxygen species
ACE2: Angiotensin converting enzyme 2
ICU: Intensive care unit
